# Use of a Multiplex PCR for the Detection of Toxin-Encoding Genes *netB* and *tpeL* in Strains of *Clostridium perfringens*

**DOI:** 10.1155/2013/865702

**Published:** 2013-12-11

**Authors:** Matthew A. Bailey, Kenneth S. Macklin, James T. Krehling

**Affiliations:** Department of Poultry Science, Auburn University, 201 Poultry Science Building, Auburn, AL 36849-5416, USA

## Abstract

Some studies have shown that the NetB toxin may be an important virulence factor of *Clostridium perfringens* associated necrotic enteritis in poultry. Additionally, research has shown that strains of *C. perfringens* positive for both the *netB* gene and a second toxin-encoding gene, *tpeL*, appear to be more virulent than strains with only *netB*. In the past, detection of these genes has been performed relatively inefficiently using two single locus PCRs. This report describes a novel multiplex PCR developed to detect *netB* and *tpeL* simultaneously in *C. perfringens* strains isolated from cases of necrotic enteritis in broilers, providing a more efficient diagnostic tool in the screening of strains for these genes.

## 1. Introduction

The disease necrotic enteritis (NE) is a major issue affecting the poultry industry, causing extensive economic loss through mortality, reduced bird performance, and carcass condemnation at slaughter [1, 2]. Caused by the bacterium *Clostridium perfringens* (CP), NE is characterized by necrotic lesions, primarily in the jejunum and ileum, which can vary in severity from thickened mucosa and multifocal ulceration in less severe cases to the formation of a greenish or yellowish pseudomembrane in the case of extensive mucosa inflammation and necrosis [3]. In the past, alpha-toxin produced by CP type A has been implicated as the primary virulence factor in NE pathogenesis [2, 4, 5]; however, the NetB toxin, encoded by the *netB* gene, was shown to be important for the virulence of certain strains [6, 7]. Another toxin, TpeL (encoded by the *tpeL* gene) [8], is a potential virulence factor as well. In a recent study, inoculation of broilers with strains positive for both *tpeL* and *netB* was associated with greater severity of gross lesions over strains with only *netB *[9].

Existing studies ascertaining the prevalence of *netB *and *tpeL* have been limited to certain geographic populations of CP, mainly in Australia, Belgium, Denmark, Sweden, and Canada [1, 7]. In the United States, studies on the prevalence of these genes have analyzed CP populations from New England, New York, and Pennsylvania [10]. The relatively small number of sampled CP populations underlines a need for more analysis to determine the importance of these genes on a worldwide scale. Additionally, detection of these genes has been performed relatively inefficiently using two single locus PCRs. This report describes a multiplex PCR developed to detect *netB* and *tpeL* simultaneously, providing a more efficient tool in screening CP strains for these genes.

## 2. Materials and Methods

Strains used as positive controls were provided by J.G. Songer of Iowa State University (JGS-1870 and JGS-4140) and the Mitchem-Sparks Regional Diagnostic Laboratory located in Boaz, Alabama (C103-99). All strains (Table 1) were prepared for DNA extraction by enriching in cooked meat medium (HiMedia Laboratories Pvt. Ltd., Mumbai, India) for 24 hr and then streaking the enriched samples for isolation onto trypticase soy agar with 5% sheep's blood (BD Diagnostics, Franklin Lakes, NJ). After 24–48 hr, a single colony exhibiting typical hemolysis was placed into brain-heart infusion (BHI) broth (BD Diagnostics) and incubated for 24 hr. All incubation was carried out at 37°C within a Bactron IV Anaerobic Environmental Chamber (Shel Lab, Cornelius, OR) with the following atmospheric conditions: 90% N_2_, 5% CO_2_, and 5% H_2_. After incubation, BHI broth cultures were centrifuged at 16,000 g for 5 min and the broth supernatant was aspirated and discarded. Utilizing a Wizard Genomic DNA Purification Kit (Promega Corp., Madison, WI), DNA was extracted from the resulting bacterial pellet. Extracted DNA was analyzed for quantity and purity with a Nanodrop 1000 Spectrophotometer (Thermo Scientific Inc., Bremen, Germany) according to the manufacturer's instructions. The standard for DNA purity was a 260/280 nm absorbance ratio of 1.8–2.0.

A previously published primer set [7] was used for the detection of *tpeL* and a primer set detecting *netB* was designed using the primer Basic Local Alignment Search Tool (BLAST) function on the National Center for Biotechnology website. Primer specifications are listed in Table 2. The *netB* primers were confirmed to target mostly conserved sequences by performing a sequence alignment with 47 *netB *sequences in GenBank using genetic analysis software.

EconoTaq PLUS GREEN Master Mix (Lucigen Corp., Middleton, WI) was used at 1x concentration providing Taq polymerase, MgCl_2_, dNTPs, and reaction buffer (pH 9.0). Reagent concentrations of the optimized multiplex were as follows: 1.2 units/25 uL of Taq DNA polymerase; 1.44 mM of MgCl_2_; 192 uM of each dNTP; 0.2 uM of each primer; and 100 ng/25 uL of template DNA. The following cycling conditions were used: an initial denaturing step at 95°C for 5 min; 40 cycles of denaturing at 95°C for 30 sec; annealing at 55°C for 30 sec, and extension at 72°C for 30 sec, with a final extension step at 72°C for 7 min.

Products were separated by electrophoresis in a 2% agarose gel. Gels and running buffer were made using 1x concentrations of AccuGENE TBE buffer (Lonza Group, Basel, Switzerland). For visualization of bands, 1 uL of an ethidium bromide solution (10 mg/mL) was added to the molten gel before solidifying. Electrophoresis took place at 100 v for about one hr or until sufficient separation between products occurred. A 100 bp DNA Ladder (Promega) was utilized as a DNA size standard. Bands corresponding to the expected amplicons were excised and DNA was purified using a GFX PCR DNA and Gel Band Purification Kit (GE Healthcare, Buckinghamshire, UK).

Forward and reverse strands of the purified DNA were sequenced by Lucigen and a consensus sequence was determined for each amplicon. These consensus sequences were then subjected to a nucleotide BLAST search using the NCBI database Nucleotide collection (nr/nt) to calculate the likelihood that each PCR product was produced from the target gene.

## 3. Results

After the analysis of 47 *netB *gene sequences published in GenBank, the target sequences for both forward (netB5F) and reverse (netB5R) primers proved to be mostly conserved. A single polymorphism was present at site number 756 of the alignment, with 21 (44.7%) of the sequences having G at this site and 26 (55.3%) having A. This site was complementary to the sixth base from the 5′ end of primer netB5R and resulted in a single A:C mismatch between the primer and 55.3% of the gene sequences. This same mutation affects primers previously described by Keyburn et al. [6], resulting in a G:T mismatch between the seventh base from the 5′ end of AKP78 (the forward primer) and 24 (51.1%) of the analyzed gene sequences.

Gel electrophoresis of PCR products confirmed the amplification of the target sequences for all three positive controls. Bands of the expected size corresponding to the *netB* (316 bp) and *tpeL* (466 bp) fragments were observed for the positive controls, and no bands were observed for the negative controls. Sequences obtained from PCR products matched the target sequence (at least 99% max identity) with significantly low *E*-values after performing a BLAST search (Table 3).

## 4. Discussion

The multiplex PCR described in this report successfully detected the *netB* and *tpeL* genes in three control organisms and did not produce false positives in the negative controls. The chance of a false negative was minimized by choosing primers that targeted mostly conserved sites within the *C. perfringens* genome. After analysis of 47 sequences, one polymorphism was observed in the target sequence indicating an internal A:C mismatch between the netB5R primer and 55.3% of the 47 aligned sequences. Although mismatches between a template and the 3′ end of a primer are known to interfere with the active site of DNA polymerase and can result in reduced reaction efficiency [11, 12], internal mismatches, or those closer to the 5′ end, have a much smaller impact [12]. In addition, out of the possible mismatches, A:C (purine-pyrimidine) mismatches generally produce only minor interferences [12]. Previously published primer AKP78^7^ contained a G:T mismatch between 51.1% of the sequences. As G:T is another purine-pyrimidine mismatch and the mismatch also occurred near the 5′ end of the primer, it is comparable to netB5R. In consequence, it is unlikely that this mismatch between the template and netB5R would have a significant impact on product formation. Although evidence suggests the *netB* and *tpeL* toxin genes may be important virulence factors for certain strains of *C. perfringens*, relatively few bacterial populations have been screened for these two genes. More populations must be screened to determine the overall impact of these genes in NE. By pairing the detection of both *netB* and *tpeL* into one PCR reaction, this multiplex PCR has the potential to increase the efficiency with which strains are screened for these two genes of interest. The effectiveness of this assay has been shown in three control organisms and must now be confirmed using a larger number of strains.

## Figures and Tables

**Table 1:** Bacterial strains used as positive and negative controls in a multiplex polymerase chain reaction designed for detection of *Clostridium perfringens* genes *netB* and *tpeL. *

Strain ID number	Species	*netB *	*tpeL *	Relevance	Source
C103-99	*Clostridium perfringens *	+	+	Positive control	Necrotic enteritis
JGS-1870	*Clostridium perfringens *	+	+	Positive control	Necrotic enteritis
JGS-4140	*Clostridium perfringens *	+	+	Positive control	Necrotic enteritis
232-2	*Clostridium bifermentans *	−	−	Negative control	Poultry litter
259-14	*Bacteroides caccae *	−	−	Negative control	Poultry litter
250-4	*Wolinella *sp.	−	−	Negative control	Poultry litter

**Table 2:** Specifications of primers used in a multiplex PCR for the detection of *netB* and *tpeL*.

Primer ID	Sequence	Target gene	Product length
netB5F	CGCTTCACATAAAGGTTGGAAGGC	*netB *	316
netB5R	TCCAGCACCAGCAGTTTTTCCT		
AKP80*	ATATAGAGTCAAGCAGTGGAG	*tpeL *	466
AKP81*	GGAATACCACTTGATATACCTG		

*Primers previously published by Keyburn et al., 2010 [7].

**Table 3:** BLAST output for sequenced PCR products*.

Target gene	Sequence source^†^	Description of top result	GenBank accession	Max identity (%)	*E*-value
*netB *	C103-99	*Clostridium perfringens* strain CP4 plasmid pCP4netB pathogenicity locus 1 genomic sequence	JF837812.1	100	5 × 10^−158^
*netB *	JGS-1870	*Clostridium perfringens *strain CP4 plasmid pCP4netB pathogenicity locus 1 genomic sequence	JF837812.1	100	3 × 10^−152^
*netB *	JGS-4140	*Clostridium perfringens *strain CP4 plasmid pCP4netB pathogenicity locus 1 genomic sequence	JF837812.1	100	1 × 10^−144^
*tpeL *	C103-99	*Clostridium perfringens *A strain CP4 TpeL (*tpeL*) gene, complete cds	EU848493.1	99	0
*tpeL *	JGS-1870	*Clostridium perfringens *A strain CP4 TpeL (*tpeL*) gene, complete cds	EU848493.1	99	0
*tpeL *	JGS-4140	*Clostridium perfringens *A strain CP4 TpeL (*tpeL*) gene, complete cds	EU848493.1	99	0

*Each row corresponds to a single sequenced band.

^†^lists the strain that the sequenced band was amplified from.
